# Human monkeypox: history, presentations, transmission, epidemiology, diagnosis, treatment, and prevention

**DOI:** 10.3389/fmed.2023.1157670

**Published:** 2023-07-20

**Authors:** Mahdi Zahmatyar, Asra Fazlollahi, Alireza Motamedi, Maedeh Zolfi, Fatemeh Seyedi, Seyed Aria Nejadghaderi, Mark J. M. Sullman, Reza Mohammadinasab, Ali-Asghar Kolahi, Shahnam Arshi, Saeid Safiri

**Affiliations:** ^1^Student Research Committee, Tabriz University of Medical Sciences, Tabriz, Iran; ^2^Neurosciences Research Center, Aging Research Institute, Tabriz University of Medical Sciences, Tabriz, Iran; ^3^Tuberculosis and Lung Diseases Research Center, Tabriz University of Medical Sciences, Tabriz, Iran; ^4^Aging Research Institute, Tabriz University of Medical Sciences, Tabriz, Iran; ^5^Systematic Review and Meta-analysis Expert Group (SRMEG), Universal Scientific Education and Research Network (USERN), Tehran, Iran; ^6^Department of Life and Health Sciences, University of Nicosia, Nicosia, Cyprus; ^7^Department of Social Sciences, University of Nicosia, Nicosia, Cyprus; ^8^Department of History of Medicine, School of Traditional Medicine, Tabriz University of Medical Sciences, Tabriz, Iran; ^9^Social Determinants of Health Research Center, Shahid Beheshti University of Medical Sciences, Tehran, Iran; ^10^Clinical Research Development Unit of Tabriz Valiasr Hospital, Tabriz University of Medical Sciences, Tabriz, Iran; ^11^Social Determinants of Health Research Center, Department of Community Medicine, Faculty of Medicine, Tabriz University of Medical Sciences, Tabriz, Iran

**Keywords:** global, epidemiology, presentations, diagnosis, treatment, prevention, monkeypox

## Abstract

Human monkeypox is a zoonotic infection that is similar to the diseases caused by other poxviruses. It is endemic among wild rodents in the rainforests of Central and Western Africa, and can be transmitted via direct skin contact or mucosal exposure to infected animals. The initial symptoms include fever, headache, myalgia, fatigue, and lymphadenopathy, the last of which is the main symptom that distinguishes it from smallpox. In order to prevent and manage the disease, those who are infected must be rapidly diagnosed and isolated. Several vaccines have already been developed (e.g., JYNNEOS, ACAM2000 and ACAM3000) and antiviral drugs (e.g., cidofovir and tecovirimat) can also be used to treat the disease. In the present study, we reviewed the history, morphology, clinical presentations, transmission routes, diagnosis, prevention, and potential treatment strategies for monkeypox, in order to enable health authorities and physicians to better deal with this emerging crisis.

## 1. Introduction

Monkeypox was previously considered to be a rare but serious zoonotic disease that is caused by the monkeypox virus, a member of the orthopoxvirus genus and close relative of the variola virus (smallpox virus) (7). The name monkeypox originates from the initial discovery of the virus in 1958, during an outbreak among monkeys in a Danish laboratory (8). Twelve years later, the first documented case in a human was identified in a 9-month-old infant in the Democratic Republic of the Congo (DRC) (9). Following this discovery, monkeypox was sporadically found in the tropical rain forests of Central and Western Africa (10), with the DRC reporting the majority of the cases each year (1, 11). The first outbreak outside of Africa occurred in 2003 (United States of America–USA), and since that time there have been several other outbreaks of the disease in non-endemic regions (12). The most recent outbreak, in 2022, surprised global public health authorities who were just starting to recover from the COVID-19 pandemic (13). The present outbreak is the largest in history, with the number of cases reported since the 7th of May 2022 having already surpassed the total number of monkeypox cases reported prior to this outbreak (14, 15). Moreover, on the 23rd of July 2022 the World Health Organization (WHO) declared the monkeypox outbreak to be a public health emergency (16).

Monkeypox produces a smallpox-like disease, but with less severe manifestations (17). Studies have shown that vaccination against smallpox can help prevent an individual from getting monkeypox (18), and can also reduce the severity of the disease (19–21). Therefore, the rapid identification and isolation of infected cases is vital, in order to prevent further transmission, protect frontline healthcare workers, and to improve clinical care (22). These goals can be achieved using a combination of different diagnostic methods, including clinical observations (3, 18) and laboratory tests (1).

As there is a need for up-to-date information on monkeypox, due to the current outbreak, we performed a comprehensive narrative review. The aim of this review was to describe the history, morphology, clinical presentations, transmission routes, diagnostic methods, prevention measures, and strategies for treating monkeypox, in order to help guide healthcare authorities and physicians in dealing with the present outbreak.

## 2. Methods

In order to identify all publications that reported important information about Monkeypox, we searched the PubMed, Scopus, and Web of Science databases. Furthermore, we also manually searched the first 300 results obtained from the Google Scholar search engine. No filters were applied to any of the search fields, such as date, study type, or language. Furthermore, in order to discover additional important studies, we performed both backward and forward citation searches of the most important articles. Our search strategy involved combining the term “monkeypox” with each of the following keywords: “epidemiology,” “presentations,” “diagnosis,” “treatment,” and “prevention.”

## 3. History

### 3.1. Overview

Monkeypox is endemic to the rainforests of Africa, although it has spread to other regions several times, leading to major outbreaks (9, 23–32). The monkeypox virus was first isolated in 1958 during an outbreak of a smallpox-like disease among cynomolgus monkeys in Copenhagen. In 1970, the first human case of monkeypox was found in a 9-month-old boy in Africa [monkeypox strain - Liberia_1970_184_DQ011156.1 (33)], via the national smallpox surveillance and eradication program (34). Following the cessation of the smallpox vaccination program in 1980, there was a decrease in herd immunity, which may have contributed to the current monkeypox outbreak (34, 35). Monkeypox is a great model for the host-pathogen interaction theory which can help find how some ancient viruses presently under investigation may have developed over time (36). In the next section, the geographical distribution and the history of monkeypox are discussed.

#### 3.1.1. Human monkeypox emerges and its geographic distribution

The monkeypox virus mostly infects wild rodents in the rainforests of Central and West Africa, with infection likely resulting from direct skin contact or mucosal exposure to infected animals (37, 38). Therefore, it is believed that people handling infected animals were the main source of the sporadic outbreaks of monkeypox (37, 38). However, the airway route plays an important role in the secondary human-to-human transmission of the virus (37, 38). The main risk factors for becoming infected with monkeypox were keeping and preparing bush meat, caring for someone infected with the virus, and not being vaccinated against smallpox (2).

**FIGURE 1:**
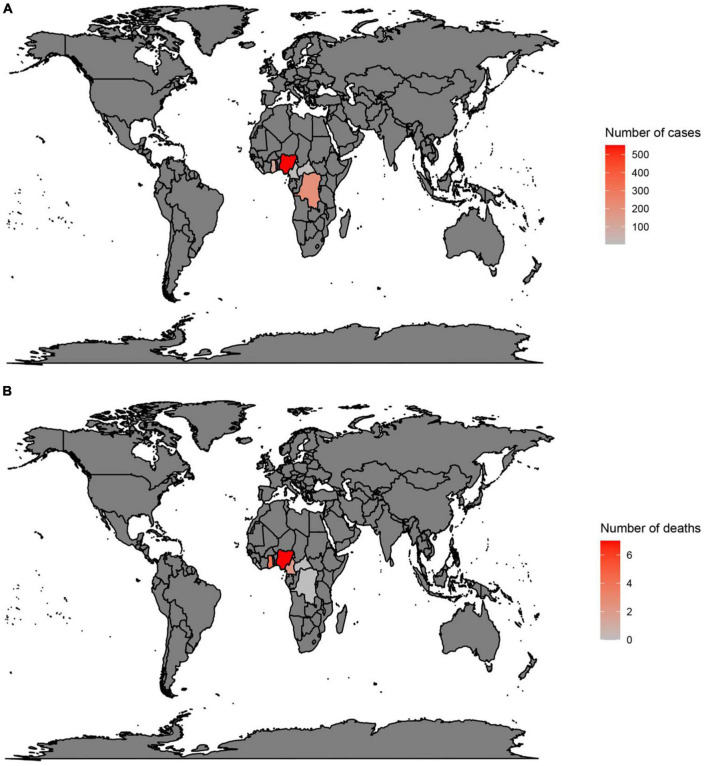
Total number of cases **(A)** and deaths **(B)** due to monkeypox in countries with a history of monkeypox. Data were retrieved from https://www.cdc.gov/poxvirus/monkeypox/response/2022/world-map.html.

**FIGURE 2:**
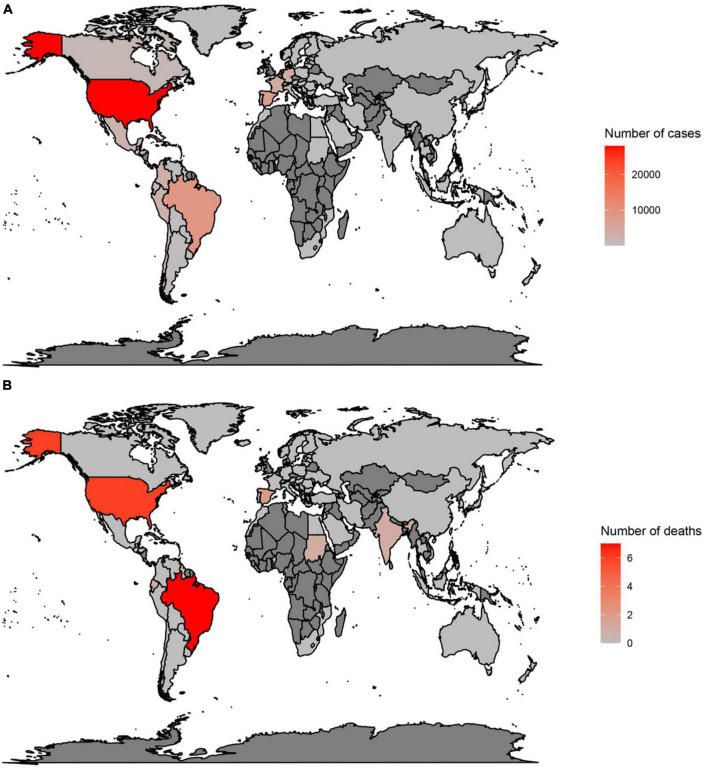
Total number of cases **(A)** and deaths **(B)** due to monkeypox, between 1 January 2022 and 24 October 2022, in countries without a history of monkeypox. Data were retrieved from https://www.cdc.gov/poxvirus/monkeypox/response/2022/world-map.html.

In countries with a history of monkeypox, the largest number of cases have been reported in Nigeria (552), the DRC (206), and Ghana (104) (Figure 1A) (3, 17, 39, 40). Moreover, deaths have been reported in Nigeria (7), Ghana (4), and Cameron (2) (Figure 1B) (3, 17, 39, 40). However, in the 2022 outbreak, from the 1st of January to the 24th of October, the largest number of cases were reported in the USA (28,004), Brazil (8,890), and Spain (7,277) (Figure 2A) (3, 17, 39, 40). Furthermore, in this same period the largest number of deaths were reported in Brazil (7), the USA (6), and Spain (2) (Figure 2B) (3, 17, 39, 40).

During the period 1970 to 2017, there were several outbreaks and sporadic infections in Central and West Africa (41). WHO recorded 54 cases in sub-Saharan Africa from 1970 to 1979, most of which occurred in the Congo (3, 17, 39, 40). However, between 1981 and 1986 WHO established an active surveillance program in the DRC, which led to the detection of 338 suspected cases (26) and 33 deaths (15, 39, 42, 43). In addition, from 1970 to 1986 there were 10 cases identified in West Africa and 394 in the Congo (Clade 1) (42).

After the surveillance program was launched by WHO, 13 cases were reported between 1986 and 1992 in Cameroon, Gabon and the DRC, but no new cases were reported between 1992 and 1995 (3, 18, 44). However, in the two following years there were large outbreaks in the Kasai-Oriental province of the DRC (3, 24, 45), in which 511 suspected cases were identified in the Katako-kombe area (24), and a further 24 cases in the Lodja Health Zone (24, 46). Following this outbreak, there were no new cases reported until 2001 (30, 39). In 2001, from February to August there were seven cases of monkeypox identified in the Equateur province of the DCR and a further four in the Mbomou province (30, 39). In total, between January 1998 and December 2002 there were 1,265 suspected cases reported to the Ministry of Health in the DCR, while over the period January 2001 to December 2004 there were 2,734 suspected cases reported in 11 provinces of the DCR (30, 39).

In 2003, the first monkeypox outbreak to occur outside the endemic regions of Africa occurred in the USA (Wisconsin, Illinois, Indiana, Missouri, Kansas, and Ohio), with 72 recorded cases (monkeypox Clade 2) (47, 48). In this outbreak, prairie dogs being transported from Ghana to Texas were infected by rodents (47, 48), and after being sold they transmitted the disease to their new owners (1, 12, 26, 29, 35, 39, 42, 46–52). This was the first monkeypox outbreak in the Western Hemisphere (3, 18) and coincided with another outbreak in the heavily forested city of Impfondo (Republic of Congo–ROC) (17). Furthermore, from September to December 2005 there were several new cases reported in Sudan’s Unity State (Clade 1) (17, 53, 54), and from 2005 to 2007 a total of 760 cases were reported from nine health zones in the DRC, which also had an active surveillance program (39). In 2010, there were a small number of new cases reported in the Likouala area, which may be due to the migration of indigenous people into the DRC (39), and two new cases in the Central African Republic (CAR) (39). Furthermore, from 2010 to 2014 there were a large number of new cases reported to the Ministry of Health in the DRC (39). In 2014, after 40 years of fighting the disease, an outbreak occurred in the Bo city area of Sierra Leone (39).

During the period September 2014 to February 2016, there were 587 cases reported in the DRC, and from December 2015 to February 2016 there were 12 cases and 3 deaths reported in Bangassou, a city in the Mbomou province of the CAR (39, 55). In August 2016, there were at least 26 cases found in the Basse-Kotto and Haute-Kotto provinces of the CAR (39), and from January to August 2017 there were 88 suspected cases reported from the Likouala province in the ROC (39). That same year there were two outbreaks in the CAR, one of which occurred in Mbomou (in February) and the other in Mbaki (in April), and in March there was another case reported in the Pujehan region of Sierra Leone (39). Furthermore, 40 years after the last outbreak of monkeypox in Liberia, an outbreak with 16 cases was reported in November and December of 2017 (39, 44).

In Nigeria, following the identification of an 11-year-old child with the disease (15, 27, 30, 39, 56–58), 244 cases was reported from September 2017 to April 2018, which was the largest monkeypox outbreak in West Africa [by monkeypox strains of Nigeria_2017_MK783029.1, Nigeria_2017_MK783028.1, Nigeria_2017_MK783032.1, Nigeria_2017_MK783031.1 and Nigeria_2017_MK783030.1 (33)] (15, 27, 30, 39, 56–58). Prior to that outbreak, only three cases had been found in Nigeria from 1970 to 2017 (one in 1970 and two in 1978) (9). However, in contrast to the DRC outbreaks, most of the cases in the Nigerian outbreak were in urban and suburban areas (59).

In the first 6 months of 2018, there were 2,845 suspected cases reported in the 14 provinces of the DRC (44). Twenty cases were found in the CAR from March 17 to April 24, and 16 cases were found in Cameroon from April 30 to May 30 (39). Unlike previous outbreaks, the 2017 Nigerian outbreak led to the spread of monkeypox into new countries and also to those living in urban areas (58). The spread of the virus into new areas was caused by the urbanization of the virus and its improved ability to infect people (49, 60).

In 2018, the cases that occurred in the United Kingdom (UK) and Israel (Clade2) can be traced back to people who had traveled to Nigeria (61, 62), and in December 2019 another case of monkeypox was found in the UK (52). That same year an infected Nigerian man traveled to Singapore (52). In fact, from September 2018 to July 2021 there were six unrelated travelers from Nigeria, who were infected with the virus. In total, 74% (144 of the 194) of the patients studied during this period were either airline passengers or were related to the six individuals who traveled from Nigeria (63–66).

**FIGURE 3:**
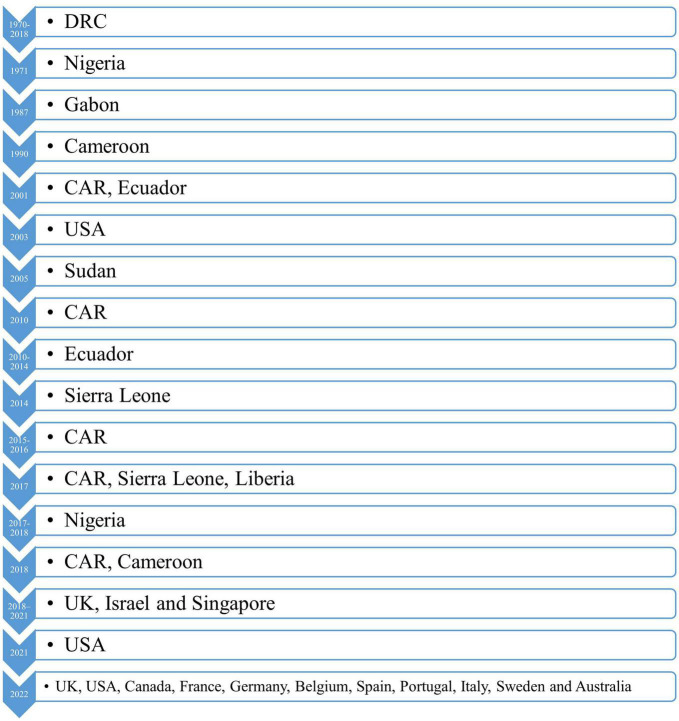
A timeline representing the outbreaks of monkeypox up to 2022. USA, United States America; UK, United Kingdom; DRC, Democratic Republic of the Congo; CAR, Central African Republic.

The 2022 monkeypox outbreak is the largest and most widespread in history, with infections being found in several European countries and the USA, raising concerns about similar future outbreaks at various ceremonies, celebrations, and festivals (67). Since the beginning of May 2022, several reports have been published in the USA, and by the end of May there were 17 cases of monkeypox in nine different states (i.e., California, Colorado, Florida, Washington, Virginia, New York, Utah, Georgia, and Massachusetts) (65). Fourteen of these patients had traveled to a different country and 16 of them were men who had sex with men (65). One of the 2022 UK cases had traveled to Nigeria, but the other two lived together and had not traveled to Nigeria nor had any contact with the first case (13, 41). Following this, outbreaks were reported in 59 countries, including Israel, the USA [strain of USA_2022_ON563414.3 (33)], Canada, Sweden, Spain [Spain_2022_ON609725.2 (33)], Italy, Germany [strain of Germany_2022_ON568298.1 (33)], Belgium, France [strain of France_2022_ON602722.2 (33)], Portugal [strain of Portugal_2022_ON585035.1 (33)], and the Netherlands. In total, there were 16016 laboratory confirmed cases identified between January 1st and July 22nd 2022 (41, 68). Moreover, the 2022 outbreak led to the first cases being found in Portugal (66) (Figure 3).

The spread of the monkeypox virus into some countries remains unclear, for instance until 16 August 2022, the monkeypox virus had not been identified in Iran (69). The Iranian Ministry of Health discovered the first case of infection in the Khuzestan province. The diagnosis of this case, who was a 34-year-old female, was confirmed through the examination of skin lesions and the subsequent genetic analysis of the virus (69). The monkeypox viruses that were identified in Iran were all part of the B.1 lineage, which originated in Europe (70). Asymptomatic carriers play a crucial role in the transmission of these viruses, so it is generally believed that monkeypox was transported into Iran from neighboring countries in the southwestern region of the country (71).

### 3.2. Classification and characterization of the monkeypox virus

The Poxviridae family consists of 22 genera and 83 species (72). The *Orthopoxvirus* genus, *which* has 12 identified members, infects both humans and animals (72). The most well-known members are the variola virus (causes smallpox), monkeypox virus, vaccinia virus (smallpox vaccine virus), and the cowpox virus (72).

The monkeypox virus has two identified clades (42). The Central African clade (Congo Basin) or Clade I causes illnesses that are similar to smallpox and has a case fatality rate of up to 10% in unvaccinated populations (72–75). Clade II or the West African Clade causes less severe illness, has lower inter-human transmissibility and has a fatality rate of 4% (72–75).

The 2022 global monkeypox outbreak reignited the debate about whether to rename the virus clades (76, 77). Consensus was reached for a third clade, that was recognized as a subset of the WA clade (clades IIa and IIb) (76–78). Finally, they renamed them Clade 1 (CB clade) and clades 2 and 3 (WA clades), to make naming less complicated (76, 77). In addition, the Clade IIb monkeypox virus recently started being classified according to their different lineages, such as A, A.1, A.1.1, A.2, and B.1, based upon single nucleotide polymorphisms and inverted terminal repeats (ITRs) (77, 79).

In some of the Nigerian cases, especially those coinfected with the human immunodeficiency virus (HIV), the West African clades have caused severe illness and even death (58, 80). Furthermore, it should be noted that the lack of medical equipment is another reason for the high morbidity and mortality found in some areas (27, 55). Moreover, population density, climate change, deforestation and animal hunting have increased the number of individuals infected (30, 61). The most common factor amongst those infected in the 2022 outbreak, was men having sex with men (64–66, 81, 82). Although the key transmission route for the present outbreak has been sexual contact between men who has sex with men, this transmission route is likely to be overlooked or hidden in countries where these types of sexual activities are illegal (83, 84).

In the 2022 outbreak, monkeypox spread widely in a number of regions and countries (39, 85). This spread was due to the virus’ ability to adapt to host immunity and the existence of suitable regions within each country for the virus to spread (39, 85). In earlier outbreaks, such as those in the 1980s, the virus was transmitted mostly through contact with animals and less commonly through human-to-human contact (3, 20, 25, 42, 86, 87). However, in the 2022 outbreak human-to-human contact was the most common mode of transmission (3, 20, 25, 42, 86, 87). This new feature of the virus raises concerns about the further spread and infection of large numbers of people (17). However, monkeypox is not as contagious as smallpox via person-to-person transmission, with only 11.7% of cases becoming infected from direct contact with patients (88).

The tumor necrosis factor receptor (TNFR, also known as J2R) and complement binding protein genes (e.g., C3L) are frequently used to distinguish between the two clades of the monkeypox virus (89). For instance, the C3L/D14L gene is often used to differentiate between clades I and II of the monkeypox virus (90), since this gene is missing in Clade II, using either the LAMP (91) or rt-PCR methods (74).

A group of researchers recently created an image database (i.e., Monkeypox Skin Lesion Dataset) for the classification of human monkeypox and other diseases like chickenpox and measles (92). The image database was created using four pre-trained models and deep learning networks, such as VGG-16, ResNet50, InceptionV3 and Ensemble (92–94).

### 3.3. Monkeypox and the eradication of smallpox

One of the most likely reasons for the increasing prevalence of monkeypox was the cessation of the smallpox vaccination programs in 1980, and the resultant decrease in herd immunity against poxviruses (34, 35, 60). Several studies have reported monkeypox to be more common in individuals who were not vaccinated against smallpox (52, 95). The smallpox vaccine could be as much as 85% effective in preventing monkeypox, so since the cessation of the smallpox immunization programs, immunity to similar orthpoxviruses (e.g., monkeypox) has also decreased (1, 15, 23, 24, 35, 39). Interestingly, in the 2000s the monkeypox virus became better able to infect older individuals and increased its ability to spread between humans (96, 97).

## 4. Transmission

Monkeypox transmission can occur through contact with body fluids, skin lesions, virus-containing waste, respiratory droplets from infected animals, and directly or indirectly through infected fomites (2, 30). Human-to-human transmission has previously been limited, but the declining herd immunity to orthopoxviruses suggests that monkeypox in humans will become more common in the future (2).

In 1980, a study of 338 monkeypox cases in the DRC found that 72.5% were thought to result from contact with animals and 27.5% from contact with other humans (97). However, in the 1990s only 22% of the 419 cases recorded in the DRC were primary infections (i.e., a person who had not reported any contact with another individual with monkeypox), while 78% were secondary infections (97). An analysis of the Nigerian outbreak data showed that transmission was unknown in 62.3% of the cases, but of those that were known 78.3% (*n* = 46) had an epidemiological association with an individual who had similar lesions and 8.2% reported having contact with animals (97).

The risk factors for monkeypox have been reported in five studies from three different countries (97). The risk factors associated with human-to-human transmission of the virus include, sleeping in a shared room or bed, living in a shared house, and drinking or eating from a shared container (97). Furthermore, sleeping outside or on the ground, living near or visiting a forest have been identified as factors that increased the risk of exposure to animals, and consequently increase the risk of animal to human transmission (97). In the 2003 USA outbreak, daily exposure to infected animals or cleaning their cages were risk factors for developing monkeypox, even among those who were vaccinated against smallpox (97).

In 1984, when research into the ecology of the smallpox virus entered its final stages, three groups of animals (i.e., rodents, squirrels, and bats) were considered priority candidates for maintaining the circulation of the virus in the wild, due mainly to their relatively high population densities (98). Following the isolation of the virus from a wild squirrel in 1985, animal samples were collected from the Bomba Zone (January to February 1986), which were later tested by WHO partner laboratories. However, no antibodies were found in any of the 233 rodents tested (98).

The high prevalence of monkeypox-specific antibodies (24.7%) that were found in 320 Funisciurus anerythrus squirrels, indicated that these animals maintained virus transmission in the areas around human habitats (98). Furthermore, the high level of antibodies found in Heliosciurus rufobrachium squirrels revealed that this species was also involved in maintaining virus transmission (98). Squirrels are obviously the main source of infection for humans, as they are the only mammals in the areas of human activity that carry monkeypox and they are frequently trapped (98). However, it is currently hard to say whether primates play an important role in maintaining the transmission of the virus in nature, or whether they are simply occasional hosts (98). The possibility of virus transmission among squirrels, outside the main areas of human activities, has also been discussed (98).

The focus of ecological studies has intensified since 1984, but the collection of samples has largely been confined to areas of the DRC with active human cases (25). These studies have led to the hypothesis that disturbed “agricultural” areas around settlements, which are rich in fungi (genera Funisciurus and Heliosciurus) and terrestrial rodents, are areas where animal contact with humans may lead to the transmission of the virus (25). In fact, the close connection between these animals and humans has greatly advanced our understanding of the virus reservoirs and the transmission of animal-to-human diseases (25).

## 5. Morphology, genome organization, and morphogenesis

Since most human cases have occurred in the CAR, at the northern edge of the western and eastern rainforests, the movement of wildlife into these rainforests is the most likely reason for the increased incidence of monkeypox in these regions (99). In addition, periods of severe political instability, which have led to an internally displaced population and increased poverty, may have increased human contact with wildlife. The socioeconomic status of the CAR, armed conflicts, and environmental disturbances are all likely to increase population mobility and human interactions with animals, thereby increasing the risk of animal-to-humans disease transmission (99). The circulation of monkeypox in human hosts, particularly among the immunocompromised, favors pathogen evolution and the emergence of new human-adapted pathogens, depending on the human pathogen fitness landscape (96).

The monkeypox virus is an enveloped brick-shaped virus that is 200–250 nanometers in diameter (56, 100). It is comprised of a dumbbell shaped biconcave core and lateral bodies that are surrounded by a corrugated lipoprotein outer membrane (56, 101). It contains linear double stranded DNA of about 197 kb (102, 103).

All orthopoxviridae genomes have a central coding region sequence at the nucleotide positions of 56,000–120,000, which is highly conserved and flanked by inverted terminals repeats (ITRs), which are identical but oppositely oriented (104). The ITRs include short tandem repeats and terminal hairpins (56). The monkeypox virus contains genes located at the terminals that encode immunomodulatory, virulence and host range factors (102, 103, 105). These genes are the main difference between the monkeypox virus and other orthopoxviridae, as well as between the different geographic strains of the monkeypox virus (102, 103, 105). The central region of the monkeypox genome contains nucleotide sequences that are the same in all monkeypox viruses and encode all essential enzymes and structural proteins, as well as the housekeeping functions (56, 102).

The poxvirus genome encodes all necessary proteins for replication and transcription (2, 106). The monkeypox virus genome contains genes that encode 190 largely non-overlapping open reading frames (ORFs) >60 amino acids or ORFs >180 nt long, which replicate in the cytoplasm (102, 103) and provide the materials needed for replication (2, 106). The ITRs region contains at least four ORFs (104). Furthermore, the genome encodes a complement-binding protein with three short consensus repeats, which contrasts to the four repeats found in other Orthopox viruses (102).

According to genome sequencing, two monkeypox virus clades can be distinguished, which are the West African and the Congo Basin (Central African) variants (67, 82). In the 2022 outbreak, the first genome sequencing was undertaken in Portugal and the results suggested that the virus was most likely from the West African clade, although some mutations were identified in the viral proteins A24R (i.e., responsible for forming a crystal structure) and H3L (i.e., important in host immune recognition) (67, 107). Furthermore, genome sequencing from Belgium and the USA confirmed that their outbreaks also originated from the West African clade (67). Phylogenetic relatedness has also been shown between the new clades and the monkeypox virus genome in the National Center for Biotechnology Information (NCBI) database (108). In an Israeli study, they found that the Israeli clade genome differed from the West African clade (Nigeria-SE-1971) by 470 single nucleotide polymorphisms (109). The recent monkeypox virus outbreak has also been linked to the monkeypox outbreaks in 2018 and 2019, which came from Nigeria to the UK, Israel, and Singapore (110). The virus structural organization is presented in the previous publication (34).

During the period 2017 to 2022, the monkeypox viruses appear to have undergone a continuous microevolution through point mutations in its genes and in multiple proteins over time, that has been observed in the available sequence data (79, 111). Several studies have reported a potential correlation between the lineage or clade and variations in the pathology of the human monkeypox disease, as well as its ability to cause outbreaks (111).

The two monkeypox clades sharing 170 orthologs and at the protein level they were found to be 99.4% identical (73, 112). Furthermore, no significant differences in the transcription regulatory sequences were found between the two genomes (73, 112). Fifty-three of the 56 virulence genes were found in both clades, with 61 conservative, 93 non-conservative, and 121 silent amino acid mutations (73). These mutations are located in the gene orthologs, such as WA clade-specific *COP-A49R* (unknown function) and *COP-A52R* (Bifunctional Toll-IL-1-receptor protein), CA clade-specific orthologs such as *BR-19* and *BR-20* (unknown functions) and other mutations, such as *BR-203* (virulence protein), *BR-209* (IL-1β binding protein), and *COP-C3L* (inhibitor of complement enzymes) (42, 73, 112). The *D14R* gene-coded inhibitor of complement-binding protein, which is an important anti-inflammatory factor, is absent from the WA clade (73, 113, 114). This gene is also responsible for the difference in virulence between the virus clades (73, 113, 114).

There are many more candidate genes that are responsible for virulence, but are yet to be identified (115). There is a sequence database^1^ that collects compiled versions of the genomes (115). Genome analysis suggest there is a very strong bias in mutations of bases guanine to adenine and cytosine to thymine (115). The Apolipoprotein B mRNA editing enzyme, which is a cytidine deaminase, is responsible for these mutations (116). A genomic comparison of MPXVgp021: L124S and MPXVgp103: K606E and other viral isolates from 2015 to 2022 showed a 30-T base long sequence in the middle of the viral genome and high frequency amino acid mutations present in the Nigeria-MPXVoutbreak-2017–2018 viruses (79, 117). These findings collectively suggest that alterations in a relatively small number of genes may contribute to the modifications in viral clearance and pathogenesis that have been observed (42, 47, 111, 118). As a result, genomic surveillance has been vital for the early detection of mutations, monitoring of virus evolution and evaluating the degree of similarity between the circulating viruses (111).

## 6. Clinical presentations and complications

### 6.1. Pathophysiology

Monkeypox causes rashes of different intensities in different species of primates (100). After entering through the available routes (oropharyngeal, nasopharyngeal or intradermal), the virus multiplies in these places and then spreads to the local lymph nodes (2). Primary viremia then leads to viral dissemination and the seeding of the virus in other organs (2). This represents the incubation period, which usually lasts for 7 to 14 days, with a maximum of 21 days (2).

### 6.2. Signs and symptoms

The onset of symptoms is associated with secondary viremia, leading to one to 2 days of prodromal symptoms (e.g., fever and lymphadenopathy) before lesions appear (2). Infected patients may have the ability to transmit the disease at this time. Lesions start in the oropharynx, before appearing on the skin. However, serum antibodies are often detectable before the lesions appear (2).

The initial symptoms of monkeypox include fever, headache, myalgia, fatigue, and lymphadenopathy, the last of which is the main symptom that distinguishes it from smallpox (1). After one to 2 days, mucosal lesions develop in the mouth followed by centrifugally distributed skin lesions on the face and limbs, including the palms of the hand and soles of the feet (1). The rash may or may not spread to the rest of the body, and the total number of lesions can range from a few to thousands (1). Over the next 2–4 weeks, the lesions evolve through the macular, papular, vesicular, and pustular phases (112). The lesions change simultaneously and are characterized as firm, deep and 2 to 10 mm in size (112). They remain in the pustular phase for five to 7 days before crusting. The crusts are formed and sloughed over the next 7 to 14 days, and in most cases this condition resolves three to 4 weeks after the onset of symptoms (112). Apart from scarring and possible skin discoloration, most patients completely recover within 4 weeks of symptom onset (39). After all the crusts disappear, the patients are no longer considered infectious (112).

The signs, symptoms, and course of the disease have been found to differ substantially, depending on whether or not the patients had been vaccinated against smallpox (119). Pleomorphism and “cropping” occurred in 31% of vaccinated patients and 18% of those who were not vaccinated (119).

It appears that the ongoing epidemic is different from previous outbreaks, in terms of the age of those affected, sex/gender (most cases have been male), risk factors, and the method of transmission, with sexual transmission being the most common means of infection (120). The most common symptom was a fever (54.29% of cases), followed by inguinal lymphadenopathy (45.71%), and exanthema (40.00%) (120). Asthenia and fatigue paired with a headache were reported in 22.86 and 25.71% of the subjects, respectively. Furthermore, myalgia and genital/anal lesions were present in 17.14 and 31.43% of the cases, respectively (120). Finally, cervical lymphadenopathy was identified in 11.43% of the patients, while the least commonly reported symptoms were diarrhea (5.71%) and axillary lymphadenopathy (5.71%) (120). Furthermore, an international study on 528 cases of monkeypox, undertaken between April and June 2022, found the incubation period to be 7 days and that the most common presentations of the disease were a rash (95%), anogenital lesions (73%), fever (62%), and lymphadenopathy (56%) (121). The different forms of lesions of monkeypox during the course of the disease is presented in the previous publication (78).

### 6.3. Prognosis

The West African variant has a more favorable prognosis, with a case fatality rate of less than 1%. The central basin (Central Africa) variant is more deadly, with a mortality rate of up to 11% in unvaccinated individuals (39). However, the prognosis is largely dependent upon the presence of severe complications (119). There have been no deaths reported among vaccinated patients, but in unvaccinated patients the crude mortality rate has been reported to be 11% (119).

### 6.4. Complications

The complications associated with monkeypox include: secondary bacterial skin infections (most common complication) or soft tissue infections (19%); permanent skin scarring, hyperpigmentation or hypopigmentation; eye complications (4–5%), such as permanent corneal scarring and vision loss; pneumonia (12%); dehydration; and sepsis (18, 30, 122).

Severe complications and sequelae, such as bronchopneumonia, sepsis, ocular infection, and neurological manifestations, have been observed more frequently among unvaccinated patients than among those who were vaccinated (1). The severity of these complications depends on the patient’s baseline health status, route of exposure, and the strain of the virus (74, 123).

Lymphadenopathy is a key manifestation that differentiates monkeypox from smallpox and other viral rash illnesses, such as chickenpox and syphilis (82, 124–128). Conjunctivitis occurred in approximately 20% of patients in a recent outbreak in the DRC. This could also be a potential site for virologic seeding into the central nervous system (86). Periocular involvements can also occur, including corneal ulcers, conjunctivitis, lesions on the eyelids, blepharitis, and even keratitis or other complications that can lead to corneal scarring and permanent vision loss or blindness (129–132). Several other complications have been reported, including: severe dehydration, caused by vomiting or diarrhea in the second week of the infection (127); skin lesions, which are characteristic with uniform progression from macules to papules, vesicles, pustules, umbilication, crusting, and desquamation (78); and extracutaneous complications (e.g., gastrointestinal symptoms) (11).

Serious complications can lead to a poor prognosis and even death. Encephalitis, acute renal injury, myocarditis, hepatomegaly and scrotal edema are some of the more serious complications (58, 108).

Serious neurologic complications from monkeypox are not common, however, headache is a common presentation in both clades 1 and 2 (58, 86). Mood disturbances, including depression and anxiety, and neuropathic pain are common (7, 80), but more serious mental health issues (e.g., suicide) are rare (133). Monkeypox can also cause encephalitis, seizures, and confusion, which have been found in about 2% of cases (134).

There have been a few cases of rectal wall perforations and abscesses in patients with proctitis, as well as myocarditis, epiglottitis, peritonsillar abscess, and hemophagocytic lymphohistiocytosis (82, 124–128, 130, 135, 136). Furthermore, rectal pain or pain on defecation (14–36% of cases), dysphagia (5–14%), inflammation of the penis (8–16%), and secondary bacterial infections (3–4%) are less severe but more common complications that have been reported in the 2022 outbreak (30, 122, 127, 136–138).

#### 6.4.1. Skin

Monkeypox can lead to skin and/or soft-tissue involvement: painful pustular lesions, genital ulcers, hyper- or hypopigmented atrophic scars, patchy alopecia, hypertrophic skin scarring, and pruritus, as well as petechiae and ulceration (32, 80, 112). Secondary bacterial skin or soft-tissue infections were found in 19% of unvaccinated monkeypox patients (1, 18). Maculopapular skin lesions of 2–5 mm in diameter typically progress through the papules, vesicles, pustules phases over a period of 2–4 weeks (18, 112). Furthermore, a pitted scar is the most common long-term consequence of infection for those who survive (119). This clinical progress is very similar to that of ordinary smallpox lesions (3). However, lesions on the mucous membranes (i.e., mouth, tongue, and genitalia) can be helpful in the differential diagnosis of monkeypox (1, 3, 139).

If the monkeypox rashes occur around the genitals, anus and bilateral inguinal areas, it is prone to the appearance of secondary bacterial cellulitis (131). In addition, when the number of skin lesions exceeds 4,500, septicemia or sepsis can also occur (139). Furthermore, monkeypox is capable of damaging other mucous membranes, which can lead to difficulty in eating or drinking, and eventually oral ulcers, pharyngitis, tonsillitis and epiglottis (131).

#### 6.4.2. Gastrointestinal system

Gastrointestinal signs are common (140). Vomiting or diarrhea can occur in the second week of the symptoms onset, which can lead to severe dehydration (1).

#### 6.4.3. Central nervous system

Encephalitis has been observed in one patient [or in less than 1% of patients (18)] and septicemia was found in a patient with more than 4,500 lesions (119, 140).

#### 6.4.4. Eyes

Ocular complications may occur in 4–5% of cases (18), and can lead to corneal ulceration and permanent vision loss (17). Corneal ulceration has been seen in 4% of those who were not vaccinated against smallpox and 1% of those who were vaccinated (29, 141).

#### 6.4.5. Other

Patients with pulmonary distress or bronchopneumonia have been observed, often late in the course of the disease, indicating a secondary infection of the lungs (119). Pneumonitis has been observed in 12% of patients (18), while bronchopneumonia has been found in 5% of those who were vaccinated against smallpox and 12% of those who were unvaccinated (29).

### 6.5. Sociodemographic characteristics

It seems that any age group or gender can develop monkeypox, if they come into contact with the virus (142). However, the overall incidence of the disease has been higher in males than in females (82, 86, 143). Furthermore, the median age of the confirmed or probable cases in the 2017–2018 Nigerian outbreak was 29 years old (range 2 days to 50 years), and 69% were male (58).

In the Tshuapa Province of the Democratic Republic of the Congo, over the period 2011–2015, the highest incidences of monkeypox were found in males aged 5–9 and 10–19 years old, followed by females aged 20–29 years old (86). As a behavioral risk factor for exposure to pathogens, 20–29 year-old women would be of childbearing age and possibly most at risk of exposure when caring for sick children (86). These females were also more likely to have contact with dead animals, which were purchased for their meat (86). Similarly, males aged 10–19 years old were likely to have more exposure to animals and to be involved in hunting (86, 143). In the Sankuru Province (DRC), the median age of patients increased from 4.4 years old in the 1980s (129, 140) to 11.9 years old during the period 2006–2007 (35). Furthermore, in the Tshuapa Province (DRC) the median age was 14 years old during the period 2011–2015 (86), and 29 years old in Nigeria during the period 2017–2018 (58).

In the 2022 outbreak, the key routes for virus transmission included having multiple sexual contacts without condoms, and having close physical contact in sexual networks (82). In this outbreak, most infected individuals were men who had sex with other men (MSM), people with multiple sexual partners and people who practice sex without a condom (82). This is also confirmed by the fact that most of the lesions have been found in the anal and genital areas (82).

Infected people or animals can be living in both urban and rural areas. In the 2017 outbreak, most of the cases in Nigeria were reported from urban and peri-urban parts of the southern regions of the country, but in the DRC most cases were from forested villages (59).

### 6.6. Mortality and morbidity

Generally, the mortality rate from monkeypox is lower than that of smallpox (1). During the 2005 conflict in Sudan, the first cases of monkeypox were reported in an arid area of the Savannah, which is quite different from the former endemic regions, which were forested, hot, humid, and often rural (53, 54, 85, 142, 144). In 2017, an outbreak in the Likouala province (ROC) led to six deaths, with a case fatality rate (CFR) of 6.8% (39). This outbreak was the largest reported in the ROC, and had a high transmission potential (39).

During the Nigerian outbreak, between 11 October 2017 and 16 September 2018, seven people (6%) died of monkeypox (3, 57, 58), four of which were also infected with HIV (58, 66). There were no deaths reported in either of the 2017 the CAR outbreaks (39). Two global studies, conducted between September 2017 and April 2018, reported a total of six deaths and a mortality rate of 2.5% (39). However, in the 2017 Liberian outbreak there were two fatalities reported, with a CFR of 12.5% (39). In addition, during the first 6 months of 2018 an outbreak in the DRC resulted in 36 deaths and a mortality rate of 1.3% (44). In contrast, the 2018 outbreaks in the CAR and Cameroon did not result in any fatalities (39).

The overall mortality rate during the African floods has been reported as being between 4 and 22% (24, 47). In contrast to the outbreaks in the Congo Basin, adults were more frequently infected in the American outbreaks of 2003 and 2017–2018 (24, 139). In general, the mortality rate in the Central and West African variants have been around 3.6 and 10.6%, respectively (5, 46, 97, 144, 145). However, recent studies have reported the CFR of the West African clade to be about 1%, but was higher in immunocompromised individuals (1, 30, 58).

As previously mentioned, there were no fatalities in the earlier outbreaks that occurred outside Africa, but from 1 January to 4 July 2022 the current outbreak caused three deaths (66, 67).

## 7. Diagnosis

### 7.1. Clinical picture

As there are strong similarities in the clinical features of monkeypox, smallpox and chickenpox, making a definitive diagnosis is important to ensure the implementation of appropriate interventions, for keeping the disease under control and preventing further transmission (3). Table 1 compares the clinical features of monkeypox, smallpox and chickenpox, which can be used to make a differential diagnosis. Although diseases such as syphilis, herpes simplex and chancroid can produce skin lesions that are similar to those observed in monkeypox, they can easily be distinguished from monkeypox using an electron microscopy (3, 9, 140, 146).

**TABLE 1:** Clinical features of monkeypox, smallpox and chickenpox.

Variable	Monkeypox	Smallpox	Chickenpox
**Time period (days)**			
Incubation stage	7–17	7–17	10–21
Prodromal stage	1–4	1–4	0–2
Illness stage (from the appearance of rashes to desquamation)	14–28	14–28	10–21
**Severity of symptoms**			
Prodromal fever	Moderate	Severe	None or mild
Fever	Moderate	Severe	Mild
Malaise	Moderate	Moderate	Mild
Headache	Moderate	Severe	Mild
Lymphadenopathy	Moderate	None	None
**Lesions**			
Distribution	Centrifugal	Centrifugal	Centripetal
Frequency of lesions on the palms or soles	Common	Common	Rare
Appearance	Hard, well-circumscribed, umbilicated	Hard, well-circumscribed, umbilicated	Superficial, irregular borders, “dew drop on a rose petal”
Depth (diameter in mm)	Deep (4–6)	Deep (4–6)	Superficial (2–4)
Evaluation	Homogenous	Homogenous	Heterogeneous
Progression	Slow progression with each stage lasting 1–2 days	Slow progression with each stage lasting 1–2 days	Fast progression
**Extracutaneous manifestations**			
Secondary skin/soft-tissue infection	19%	Possible	Possible
Pneumonitis	12%	Possible	3–16%
Ocular complications	4–5%	5–9%	No
Encephalitis	<1%	<1%	<1%

Clinically, the skin eruptions of monkeypox are almost identical to those seen in the ordinary forms of smallpox, and are also similar to both classic chickenpox and atypical chickenpox (3, 9, 140, 146). In addition, the histologic features of monkeypox are very similar to those of smallpox and cowpox, although they are both noticeably different from other pox viruses (56, 147). The crop-like, less centrifugally distributed lesions (than smallpox), and especially the presence of lymphadenopathy may indicate monkeypox (56), as lymphadenopathy is not a common feature of smallpox (95). Lymphadenopathy in the submandibular and the cervical or inguinal regions are also considered to be a key diagnostic feature that distinguishes monkeypox from smallpox (3, 148).

### 7.2. Laboratory testing

Although clinical characteristics can be helpful in differentiating monkeypox from other infectious causes of vesiculopustular rashes, laboratory confirmation is necessary to make a definitive diagnosis (149). Several techniques are available for diagnosing monkeypox, such as serology, electron microscopy, and an enzyme-linked immunosorbent assay (ELISA), which are discussed later in this section (149).

#### 7.2.1. Histology

The examination of skin biopsy specimens from patients with monkeypox shows central necrosis surrounded by hyperplastic epidermis, spongiosis and ballooning degeneration of the keratinocytes (150). An inflammatory infiltration, which is composed of lymphocytes, eosinophils and neutrophils, is evident in the epidermis and the superficial layers of the dermis (150). Multinucleated giant cells and eosinophilic inclusion bodies can be observed in the different layers of the epidermis (150). Follicular involvement and dyskeratotic keratinocytes can also be seen in the follicular epithelium (150). The histopathologic findings of monkeypox are non-specific and are highly similar to other infectious viral processes, such as smallpox, varicella zoster, herpes simplex, vaccinia and cowpox (150).

#### 7.2.2. Immunohistochemistry

Immunohistochemical staining can help to distinguish a poxvirus from a herpesviruses (123). There are some polyclonal antibodies available (e.g., anti-vaccinia murine) that have high potency in the detection of the orthopoxvirus, but do not cross-react with the herpes simplex virus (123). Furthermore, antibodies against herpes simplex virus are available, which do not cause any cross-reactions with the monkeypox virus (150). Monoclonal antibodies against the monkeypox virus can detect the presence of the orthopox antigen and can even recognize it as the monkeypox virus (151).

Orthopoxvirus-specific IgG and IgM appear 5 to 8 days post-infection with monkeypox or vaccination with the vaccinia virus, and may be detected using an ELISA or a lateral flow immunochromatographic test (4, 61, 123, 150, 152). However, these antibodies cannot differentiate between the various orthopoxviruses, as a result of cross-reactivity (61, 123, 150, 152). Additionally, IgM is more specific than IgG, since IgG can be positive due to past exposure or having been vaccinated against smallpox, due to the long-term persistence of residual IgG-memory B cells (61, 123, 150, 152).

#### 7.2.3. Polymerase Chain Reaction (PCR)

The Polymerase Chain Reaction (PCR) technique, including real-time PCR evaluation of a specimen, can be used to detect the presence of monkeypox specific DNA (153–156). These methods are highly sensitive and the real-time PCR approach is currently the best diagnostic assay for the detection of monkeypox (153–156).

## 8. Prevention

As close contact with an infected person is the main risk factor for the transmission of the disease (65), wearing face masks and hygienic hand washing can play an important role in preventing disease transmission (65). In this context, wearing an N95 mask is more effective in preventing transmission than using a surgical mask (157). Healthcare workers should use highly protective personal equipment when in contact with an infected patient, due to the high virus titers present in pus and scabs, which result in an increased risk of human-to-human transmission (61). Moreover, it is extremely important to identify and isolate people who have had sexual relationships with infected individuals or who have traveled to areas with cases of monkeypox (5, 65). It is recommended that preventive procedures should be kept in place for 4 to 14 days after exposure (61).

The most reliable approach in dealing with infected subjects is to isolate them until their lesions are fully healed, and for long-term hospitalization, local health authorities and hospitals must plan appropriately (158). Initiating education campaigns to increase public awareness of the disease could also be helpful in several parts of the world (158). Educational interventions can play an important role in reducing the incidence of high-risk behaviors, familiarizing the population about the symptoms of the disease, and increasing the timely referral of oneself or family members to the hospital, if symptoms are observed (159).

Vaccination against monkeypox appears to be more difficult than it was for smallpox, as monkeypox is transmitted by both animals and humans, while smallpox was only transmitted by humans (6, 23). Nevertheless, vaccines have an important role to play in preventing monkeypox, and can even be used as a post exposure prophylaxis (PEP) (160). The ACAM2000 and JYNNEOS vaccines can both be given to those at high-risk of occupational exposure, or those who may have been exposed to the monkeypox virus (6). These vaccines have been safely used by public health authorities in the USA, the UK, and Singapore (61).

ACAM2000, which was the only vaccine recommended as a PEP (5), has been associated with some cardiac complications (12, 18). The Centers for Disease Control and Prevention (CDC), WHO and the Advisory Committee on Immunization Practices (ACIP) do not recommend pre-event vaccination, except for a small number of identified groups, including field researchers, veterinarians, infected animal controllers, military personnel, laboratory and healthcare workers (12, 18, 161).

People with immunodeficiency are recommended to use intravenous immunoglobulin (IVIG) therapy, although the benefits have not yet been properly evaluated (5, 50, 162, 163). The third generation Modified Vaccinia virus Ankara (MVA), an attenuated strain of the vaccinia virus, can be used in two doses with 4-week intervals. Unlike vaccines made from a live virus, MVA does not cause skin lesions (1, 2). Clinical trials have shown that this vaccine can stimulate the production of antibodies and that it can be used in immunocompromised patients and individuals with contraindications to live virus vaccines (50) (Table 2). Collectively, the prevention of future monkeypox outbreaks should involve reducing global zoonosis infections, population-level surveillance, shutting down transmission chains, as well as designing and promoting adequate vaccines and antivirals (164).

**TABLE 2:** Characteristics of the different potential medicines for the treatment or prevention of monkeypox.

Name	Dose and consumption instructions	Effectiveness
ACAM2000 vaccine	N/A	Highly protective against monkeypox infection and is also recommended within 4 days of having close contact with a confirmed monkeypox case
Cidofovir	Administered intravenously and accompanied by probenecid and hydration (avoiding renal toxicity).	It is effective against many DNA viruses, including monkeypox.
Tecovirimat SIGA (Tpoxx) or Brincidofovir	N/A	This medicine is used to deal with orthopoxvirus infections.
ST-246 (Drug)	Oral administration	It prevents the intracellular spread of the virus and is used in orthopoxvirus infections.
CMX-001	N/A	A modified formulation of cidofovir that lacks the same degree of nephrotoxicity.

N/A, not available.

## 9. Treatment

There is currently no specific therapy for treating the underlying cause of this disease (2, 30, 158). Therefore, symptomatic treatment and the prevention of secondary infections are the recommended approaches (2, 30, 158). At the time of this review, there was no definitive evidence that antivirals were effective for treating monkeypox in humans, but since they are effective in treating animals, they are also expected to be effective in treating humans (6, 165, 166). Although there are no monkeypox-specific antivirals, antivirals such as brincidofovir and tecovirimat can be used to treat monkeypox (5, 167). Tecovirimat is safe to be used in the early stages of the infection, and if treatment is initiated during the incubation period it can prevent overt clinical presentations (168, 169). Brincidofovir, which is a lipid conjugation of cidofovir, can also be used to treat monkeypox, although in some cases it has resulted in elevated liver enzymes (7). The duration of treatment is around 14 days (170) (Table 2).

## 10. Conclusion

The 2022 monkeypox outbreak has deeply affected the world’s healthcare systems, which were simultaneously combating the COVID-19 pandemic. The expansion of the disease outside Africa indicates that it is no longer a rare viral infection that is only found in the forested regions of Central and Western Africa. The outbreak has again highlighted the importance of early diagnostic and preventive methods in effectively tackling the spread of a new and highly transmissible disease. Therefore, it is essential to identify the clinical features of the disease, to determine appropriate diagnostic methods, as well as to develop techniques to effectively treat and prevent this disease.

## Ethics statement

This study was reviewed and approved by Ethics Committee of Shahid Beheshti University of Medical Sciences, Tehran, Iran (IR.SBMU.RETECH.REC.1401.389).

## Author contributions

SS, SA, MZ, SAN, and A-AK designed the study. MZ, AF, AM, MZo, FS, SAN, MJMS, RM, A-AK, SA, and SS drafted the initial manuscript. All authors reviewed the drafted manuscript for critical content and approved the final version of the manuscript.

## Footnotes

1
https://nextclade.org/monkeypox

## References

[1] McCollum A Damon I. Human monkeypox. *Clin Infect Dis.* (2014) 58:260–7. 24158414 10.1093/cid/cit703 PMC5895105

[2] Moore M Zahra F. *Monkeypox.* Treasure Island, FL: StatPearls Publishing Copyright (2022).

[3] Weinstein R Nalca A Rimoin A Bavari S Whitehouse C. Reemergence of monkeypox: prevalence, diagnostics, and countermeasures. *Clin Infect Dis.* (2005) 41:1765–71. 16288402 10.1086/498155

[4] Osborn L Villarreal D Wald-Dickler N Bard J. Monkeypox: clinical considerations, epidemiology, and laboratory diagnostics. *Clin Microbiol Newslett.* (2022) 44:199–208. 10.1016/j.clinmicnews.2022.11.003 PMC9674185 36438980

[5] Costello V Sowash M Gaur A Cardis M Pasieka H Wortmann G Imported monkeypox from international traveler, Maryland, USA, 2021. *Emerg Infect Dis.* (2022) 28:1002. 10.3201/eid2805.220292 PMC9045429 35263559

[6] Afshar Z Rostami H Hosseinzadeh R Janbakhsh A Pirzaman A Babazadeh A The reemergence of monkeypox as a new potential health challenge: a critical review. *Authorea.* [Preprint] (2022) 10.22541/au.165446104.43472483/v1

[7] Adler H Gould S Hine P Snell L Wong W Houlihan C Clinical features and management of human monkeypox: a retrospective observational study in the UK. *Lancet Infect Dis.* (2022) 22:1153–62. 10.1016/S1473-3099(22)00228-6 35623380 PMC9300470

[8] Magnus P Andersen E Petersen K Birch-Andersen A. A pox-like disease in cynomolgus monkeys. *Acta Pathol Microbiol Scand.* (2009) 46:156–76.

[9] Breman J Kalisa-Ruti M Zanotto E Gromyko A Arita I. Human monkeypox, 1970-79. *Bull World Health Organ.* (1980) 58:165. 6249508 PMC2395797

[10] Fine P Jezek Z Grab B Dixon H. The transmission potential of monkeypox virus in human populations. *Int J Epidemiol.* (1988) 17:643–50. 2850277 10.1093/ije/17.3.643

[11] Parker S Nuara A Buller R Schultz D. Human monkeypox: an emerging zoonotic disease. *Future Microbiol.* (2007) 2:17–34. 17661673 10.2217/17460913.2.1.17

[12] Brown K Leggat P. Human monkeypox: current state of knowledge and implications for the future. *Trop Med Infect Dis.* (2016) 1:8. 10.3390/tropicalmed1010008 30270859 PMC6082047

[13] Zumla A Valdoleiros S Haider N Asogun D Ntoumi F Petersen E Monkeypox outbreaks outside endemic regions: scientific and social priorities. *Lancet Infect Dis.* (2022) 22:929–31. 10.1016/S1473-3099(22)00354-1 35636447 PMC9141675

[14] World Health Organization [WHO]. *Disease Outbreak News; Monkeypox– United Kingdom of Great Britain and Northern Ireland.* Geneva: World Health Organization (2022).

[15] Petersen E Abubakar I Ihekweazu C Heymann D Ntoumi F Blumberg L Monkeypox - Enhancing public health preparedness for an emerging lethal human zoonotic epidemic threat in the wake of the smallpox post-eradication era. *Int J Infect Dis.* (2019) 78:78–84. 10.1016/j.ijid.2018.11.008 30453097 PMC7129336

[16] Nuzzo J Borio L Gostin L. The WHO declaration of monkeypox as a global public health emergency. *JAMA.* (2022) 328:615–7. 35895041 10.1001/jama.2022.12513

[17] Learned L Reynolds M Wassa D Li Y Olson V Karem K Extended interhuman transmission of monkeypox in a hospital community in the Republic of the Congo, 2003. *Am J Trop Med Hyg.* (2005) 73:428–34. 16103616

[18] Di Giulio D Eckburg P. Human monkeypox: an emerging zoonosis. *Lancet Infect Dis.* (2004) 4:15–25. 14720564 10.1016/S1473-3099(03)00856-9 PMC9628772

[19] Hammarlund E Lewis M Carter S Amanna I Hansen S Strelow L Multiple diagnostic techniques identify previously vaccinated individuals with protective immunity against monkeypox. *Nat Med.* (2005) 11:1005–11. 10.1038/nm1273 16086024

[20] Ježek Z Grab B Szczeniowski M Paluku K Mutombo M. Human monkeypox: secondary attack rates. *Bull World Health Organ.* (1988) 66:465. PMC2491159 2844429

[21] Jezek Z Marennikova S Mutumbo M Nakano J Paluku K Szczeniowski M. Human monkeypox: a study of 2,510 contacts of 214 patients. *J Infect Dis.* (1986) 154:551–5. 10.1093/infdis/154.4.551 3018091

[22] World Health Organization [WHO]. *Surveillance, case investigation and contact tracing for Monkeypox: Interim guidance.* Geneva: World Health Organization (2022).

[23] Reynolds M Damon I. Outbreaks of human monkeypox after cessation of smallpox vaccination. *Trends Microbiol.* (2012) 20:80–7. 22239910 10.1016/j.tim.2011.12.001

[24] Hutin Y Williams R Malfait P Pebody R Loparev V Ropp S Outbreak of human monkeypox, Democratic Republic of Congo, 1996 to 1997. *Emerg Infect Dis.* (2001) 7:434. 10.3201/eid0703.010311 PMC2631782 11384521

[25] Reynolds M Doty J McCollum A Olson V Nakazawa Y. Monkeypox re-emergence in Africa: a call to expand the concept and practice of One Health. *Expert Rev Anti Infect Ther.* (2019) 17:129–39. 10.1080/14787210.2019.1567330 30625020 PMC6438170

[26] Ligon B. Monkeypox: a review of the history and emergence in the Western hemisphere. *Semin Pediatr Infect Dis.* (2004) 15:280–7. 10.1053/j.spid.2004.09.001 15494953 PMC7129998

[27] Kabuga A El Zowalaty ME. A review of the monkeypox virus and a recent outbreak of skin rash disease in Nigeria. *J Med Virol.* (2019) 91:533–40. 10.1002/jmv.25348 30357851

[28] Beer E Rao VB. A systematic review of the epidemiology of human monkeypox outbreaks and implications for outbreak strategy. *PLoS Neglected Trop Dis.* (2019) 13:e0007791. 10.1371/journal.pntd.0007791 31618206 PMC6816577

[29] Damon I. Status of human monkeypox: clinical disease, epidemiology and research. *Vaccine.* (2011) 29:D54–9. 22185831 10.1016/j.vaccine.2011.04.014

[30] Durski K McCollum A Nakazawa Y Petersen B Reynolds M Briand S Emergence of monkeypox—west and central Africa, 1970–2017. *Morbidity Mortal Wkly Rep.* (2018) 67:306. 10.15585/mmwr.mm6710a5 PMC5857192 29543790

[31] Ellis C Carroll D Lash R Peterson A Damon I Malekani J Ecology and geography of human monkeypox case occurrences across Africa. *J Wildl Dis.* (2008) 48:335–47. 10.7589/0090-3558-48.2.335 22493109

[32] Fowotade A Fasuyi T Bakare R. Re-emergence of monkeypox in Nigeria: a cause for concern and public enlightenment. *Afr J Clin Exp Microbiol.* (2018) 19:307–13.

[33] Wassenaar T Wanchai V Ussery D. Comparison of monkeypox virus genomes from the 2017 Nigeria outbreak and the 2022 outbreak. *J Appl Microbiol.* (2022) 133:3690–8. 36074056 10.1111/jam.15806 PMC9828465

[34] Gessain A Nakoune E Yazdanpanah Y. Monkeypox. *N Engl J Med.* (2022) 387:1783–93. 36286263 10.1056/NEJMra2208860

[35] Rimoin A Mulembakani P Johnston S Smith J Kisalu N Kinkela T Major increase in human monkeypox incidence 30 years after smallpox vaccination campaigns cease in the Democratic Republic of Congo. *Proc Natl Acad Sci U S A.* (2010) 107:16262–7. 10.1073/pnas.1005769107 20805472 PMC2941342

[36] Galassi F Sineo L Papa V Varotto E. Monkeypox between dermatology and anthropology: a model for evolutionary medicine. *Clin Dermatol.* (2023) 10.1016/j.clindermatol.2023.04.001 [Epub ahead of print]. 37076103 PMC10110996

[37] Americo J Moss B Earl P. Identification of wild-derived inbred mouse strains highly susceptible to monkeypox virus infection for use as small animal models. *J Virol.* (2010) 84:8172–80. 10.1128/JVI.00621-10 20519404 PMC2916512

[38] Osorio J Iams K Meteyer C Rocke T. Comparison of monkeypox viruses pathogenesis in mice by in vivo imaging. *PLoS One.* (2009) 4:e6592. 10.1371/journal.pone.0006592 19668372 PMC2719101

[39] Sklenovska N Van Ranst M. Emergence of monkeypox as the most important orthopoxvirus infection in humans. *Front Public Health.* (2018) 6:241. 10.3389/fpubh.2018.00241 30234087 PMC6131633

[40] Breman J. Monkeypox: an emerging infection for humans? In: Michael Scheld W Craig WA Hughes JM editors. *Emerging infections 4.* Hoboken, NJ: Wiley (2000). p. 45–67.

[41] Shanmugaraj B Khorattanakulchai N Phoolcharoen W. Emergence of monkeypox: another concern amidst COVID-19 crisis. *Asian Pac J Trop Med.* (2022) 15:193.

[42] Likos A Sammons S Olson V Frace A Li Y Olsen-Rasmussen M A tale of two clades: monkeypox viruses. *J Gene Virol.* (2005) 86:2661–72. 10.1099/vir.0.81215-0 16186219

[43] Lash R Carroll D Hughes C Nakazawa Y Karem K Damon I Effects of georeferencing effort on mapping monkeypox case distributions and transmission risk. *Int J Health Geogr.* (2012) 11:23. 10.1186/1476-072X-11-23 22738820 PMC3724478

[44] Rimoin A Kisalu N Kebela-Ilunga B Mukaba T Wright L Formenty P Endemic human monkeypox, democratic Republic of Congo, 2001–2004. *Emerg Infect Dis.* (2007) 13:934. 10.3201/eid1306.061540 17553242 PMC2792850

[45] Heymann D Szczeniowski M Esteves K. Re-emergence of monkeypox in Africa: a review of the past six years. *Br Med Bull.* (1998) 54:693–702. 10.1093/oxfordjournals.bmb.a011720 10326294

[46] Maskalyk J. Monkeypox outbreak among pet owners. *CMAJ.* (2003) 169:44–5. 12847040 PMC164943

[47] Reed K Melski J Graham M Regnery R Sotir M Wegner M The detection of monkeypox in humans in the Western Hemisphere. *N Engl J Med.* (2004) 350:342–50. 14736926 10.1056/NEJMoa032299

[48] Gross E. Update: multistate outbreak of monkeypox—Illinois, Indiana, Kansas, Missouri, Ohio, and Wisconsin, 2003. *Ann Emerg Med.* (2003) 42:664. 10.1016/S0196-0644(03)00819-9 PMC9533866 14581919

[49] Yong S Ng O Ho Z Mak T Marimuthu K Vasoo S Imported Monkeypox, Singapore. *Emerg Infect Dis.* (2020) 26:1826. 10.3201/eid2608.191387 PMC7392406 32338590

[50] Sejvar J Chowdary Y Schomogyi M Stevens J Patel J Karem K Human monkeypox infection: a family cluster in the midwestern United States. *J Infect Dis.* (2004) 190:1833–40. 15499541 10.1086/425039

[51] Langkop C Austin C Dworkin M Kelly K Messersmith H Teclaw R Multistate outbreak of monkeypox-Illinois, Indiana, Kansas, Missouri, Ohio, and Wisconsin, 2003. *Morbid Mortal Wkly Rep.* (2003) 52:561–3.

[52] Simpson K Heymann D Brown C Edmunds W Elsgaard J Fine P Human monkeypox–After 40 years, an unintended consequence of smallpox eradication. *Vaccine.* (2020) 38:5077–81. 32417140 10.1016/j.vaccine.2020.04.062 PMC9533855

[53] Formenty P Muntasir M Damon I Chowdhary V Opoka M Monimart C Human monkeypox outbreak caused by novel virus belonging to Congo Basin clade, Sudan, 2005. *Emerg Infect Dis.* (2010) 16:1539. 10.3201/eid1610.100713 20875278 PMC3294404

[54] Reynolds M Emerson G Pukuta E Karhemere S Muyembe J Bikindou A Detection of human monkeypox in the Republic of the Congo following intensive community education. *Am J Trop Med Hyg.* (2013) 88:982. 10.4269/ajtmh.12-0758 23400570 PMC3752768

[55] Kalthan E Tenguere J Ndjapou S Koyazengbe T Mbomba J Marada R Investigation of an outbreak of monkeypox in an area occupied by armed groups, Central African Republic. *Med Maladies Infect.* (2018) 48:263–8. 10.1016/j.medmal.2018.02.010 29573840 PMC9533891

[56] Alakunle E Moens U Nchinda G Okeke M. Monkeypox virus in Nigeria: infection biology, epidemiology, and evolution. *Viruses.* (2020) 12:1257. 10.3390/v12111257 PMC7694534 33167496

[57] Rezza G. Emergence of human monkeypox in west Africa. *Lancet Infect Dis.* (2019) 19:797–9. 31285141 10.1016/S1473-3099(19)30281-6

[58] Yinka-Ogunleye A Aruna O Dalhat M Ogoina D McCollum A Disu Y Outbreak of human monkeypox in Nigeria in 2017–18: a clinical and epidemiological report. *Lancet Infect Dis.* (2019) 19:872–9. 31285143 10.1016/S1473-3099(19)30294-4 PMC9628943

[59] Ihekweazu C Yinka-Ogunleye A Lule S Ibrahim A. Importance of epidemiological research of monkeypox: is incidence increasing? *Expert Rev Anti Infect Ther.* (2020) 18:389–92. 32096659 10.1080/14787210.2020.1735361 PMC9491128

[60] Nguyen P Ajisegiri W Costantino V Chughtai A MacIntyre C. Reemergence of human monkeypox and declining population immunity in the context of urbanization, Nigeria, 2017–2020. *Emerg Infect Dis.* (2021) 27:1007. 10.3201/eid2704.203569 33756100 PMC8007331

[61] Erez N Achdout H Milrot E Schwartz Y Wiener-Well Y Paran N Diagnosis of imported monkeypox, Israel, 2018. *Emerg Infect Dis.* (2019) 25:980. 10.3201/eid2505.190076 PMC6478227 30848724

[62] Vaughan A Aarons E Astbury J Balasegaram S Beadsworth M Beck C Two cases of monkeypox imported to the United Kingdom, September 2018. *Eurosurveillance.* (2018) 23:1800509. 10.2807/1560-7917.ES.2018.23.38.1800509 30255836 PMC6157091

[63] Rao A Schulte J Chen T Hughes C Davidson W Neff J Monkeypox in a Traveler Returning from Nigeria—Dallas, Texas, July 2021. *Morbid Mortal Wkly Rep.* (2022) 71:509. 10.15585/mmwr.mm7114a1 35389974 PMC8989376

[64] Cohen J. Monkeypox outbreak questions intensify as cases soar. *Science.* (2022) 376:902–3. 10.1126/science.add1583 35617408

[65] Minhaj F Ogale Y Whitehill F Schultz J Foote M Davidson W Monkeypox outbreak—nine states, May 2022. *MMWR Morb Mortal Wkly Rep.* (2022) 71:764–9. 35679181 10.15585/mmwr.mm7123e1 PMC9181052

[66] Duque M Ribeiro S Martins J Casaca P Leite P Tavares M Ongoing monkeypox virus outbreak, Portugal, 29 April to 23 May 2022. *Eurosurveillance.* (2022) 27:2200424. 10.2807/1560-7917.ES.2022.27.22.2200424 35656830 PMC9164676

[67] Velavan T Meyer C. Monkeypox 2022 outbreak: an update. *Trop Med Int Health.* (2022) 27:604–5. 35633308 10.1111/tmi.13785

[68] World Health Organization [WHO]. *Multi-country outbreak of monkeypox, External situation report #2 - 25 July 2022.* Geneva: World Health Organization (2022).

[69] Letafati A Sakhavarz T. Monkeypox virus: a review. *Microb Pathog.* (2023) 176:106027. 10.1016/j.micpath.2023.106027 PMC9907786 36758824

[70] Luna N Ramírez A Muñoz M Ballesteros N Patiño L Castañeda S Phylogenomic analysis of the monkeypox virus (MPXV) 2022 outbreak: emergence of a novel viral lineage? *Travel Med Infect Dis.* (2022) 49:102402. 10.1016/j.tmaid.2022.102402 35840078 PMC9628808

[71] De Baetselier I Van Dijck C Kenyon C Coppens J Michiels J de Block T Retrospective detection of asymptomatic monkeypox virus infections among male sexual health clinic attendees in Belgium. *Nat Med.* (2022) 28:2288–92. 10.1038/s41591-022-02004-w 35961373 PMC9671802

[72] Karagoz A Tombuloglu H Alsaeed M Tombuloglu G AlRubaish A Mahmoud A Monkeypox (mpox) virus: classification, origin, transmission, genome organization, antiviral drugs, and molecular diagnosis. *J Infect Public Health.* (2023) 16:531–41. 10.1016/j.jiph.2023.02.003 36801633 PMC9908738

[73] Chen N Li G Liszewski M Atkinson J Jahrling P Feng Z Virulence differences between monkeypox virus isolates from West Africa and the Congo basin. *Virology.* (2005) 340:46–63. 16023693 10.1016/j.virol.2005.05.030 PMC9534023

[74] Li Y Zhao H Wilkins K Hughes C Damon I. Real-time PCR assays for the specific detection of monkeypox virus West African and Congo Basin strain DNA. *J Virol Methods.* (2010) 169:223–7. 10.1016/j.jviromet.2010.07.012 20643162 PMC9628942

[75] Sah R Abdelaal A Reda A Katamesh B Manirambona E Abdelmonem H Monkeypox and its possible sexual transmission: where are we now with its evidence? *Pathogens.* (2022) 11:924.

[76] Ulaeto D Agafonov A Burchfield J Carter L Happi C Jakob R New nomenclature for mpox (monkeypox) and monkeypox virus clades. *Lancet Infect Dis.* (2023) 23:273–5. 10.1016/S1473-3099(23)00055-5 36758567 PMC9901940

[77] Happi C Adetifa I Mbala P Njouom R Nakoune E Happi A Urgent need for a non-discriminatory and non-stigmatizing nomenclature for monkeypox virus. *PLoS Biol.* (2022) 20:e3001769. 10.1371/journal.pbio.3001769 35998195 PMC9451062

[78] Billioux B Mbaya O Sejvar J Nath A. Neurologic complications of smallpox and monkeypox: a review. *JAMA Neurol.* (2022) 79:1180–6. 36125794 10.1001/jamaneurol.2022.3491

[79] Desingu P Rubeni T Sundaresan N. Evolution of monkeypox virus from 2017 to 2022: in the light of point mutations. *Front Microbiol.* (2022) 13:1037598. 10.3389/fmicb.2022.1037598 36590408 PMC9795006

[80] Ogoina D Iroezindu M James H Oladokun R Yinka-Ogunleye A Wakama P Clinical course and outcome of human monkeypox in Nigeria. *Clin Infect Dis.* (2020) 71:e210–4. 10.1093/cid/ciaa143 32052029

[81] Heskin J Belfield A Milne C Brown N Walters Y Scott C Transmission of monkeypox virus through sexual contact–A novel route of infection. *J Infect.* (2022) 85:334–63. 10.1016/j.jinf.2022.05.028 35659548 PMC9534114

[82] Antinori A Mazzotta V Vita S Carletti F Tacconi D Lapini L Epidemiological, clinical and virological characteristics of four cases of monkeypox support transmission through sexual contact. Italy, May 2022. *Eurosurveillance.* (2022) 27:2200421. 10.2807/1560-7917.ES.2022.27.22.2200421 35656836 PMC9164671

[83] Mahase E. Monkeypox: what do we know about the outbreaks in Europe and North America? *BMJ.* (2022) 377:o1274. 10.1136/bmj.o1274 35595274

[84] Zarocostas J. Monkeypox PHEIC decision hoped to spur the world to act. *Lancet.* (2022) 400:347. 10.1016/S0140-6736(22)01419-2 35908563 PMC9534077

[85] Nakazawa Y Mauldin M Emerson G Reynolds M Lash R Gao J A phylogeographic investigation of African monkeypox. *Viruses.* (2015) 7:2168–84. 10.3390/v7042168 25912718 PMC4411695

[86] Whitehouse E Bonwitt J Hughes C Lushima R Likafi T Nguete B Clinical and Epidemiological Findings from Enhanced Monkeypox Surveillance in Tshuapa Province, Democratic Republic of the Congo During 2011–2015. *J Infect Dis.* (2021) 223:1870–8. 10.1093/infdis/jiab133 33728469

[87] Reynolds M Yorita K Kuehnert M Davidson W Huhn G Holman R Clinical manifestations of human monkeypox influenced by route of infection. *J Infect Dis.* (2006) 194:773–80. 16941343 10.1086/505880

[88] Kantele A Chickering K Vapalahti O Rimoin A. Emerging diseases—the monkeypox epidemic in the Democratic Republic of the Congo. *Clin Microbiol Infect.* (2016) 22:658–9. 10.1016/j.cmi.2016.07.004 27404372 PMC9533887

[89] Ghate S Suravajhala P Patil P Vangala R Shetty P Rao R. Molecular detection of monkeypox and related viruses: challenges and opportunities. *Virus Genes.* (2023) 59:343–50. 10.1007/s11262-023-01975-3 36746846 PMC9901828

[90] Saijo M Ami Y Suzaki Y Nagata N Iwata N Hasegawa H Diagnosis and assessment of monkeypox virus (MPXV) infection by quantitative PCR assay: differentiation of Congo Basin and West African MPXV strains. *Jap J Infect Dis.* (2008) 61:140. 18362406

[91] Iizuka I Saijo M Shiota T Ami Y Suzaki Y Nagata N Loop-mediated isothermal amplification-based diagnostic assay for monkeypox virus infections. *J Med Virol.* (2009) 81:1102–8. 10.1002/jmv.21494 19382264

[92] Ali S Ahmed M Paul J Jahan T Sani S Noor N Monkeypox skin lesion detection using deep learning models: a feasibility study. *Arxiv.* [Preprint]. (2022) 10.48550/arXiv.2207.03342

[93] Sahin V Oztel I Yolcu Oztel G. Human monkeypox classification from skin lesion images with deep pre-trained network using mobile application. *J Med Syst.* (2022) 46:79. 10.1007/s10916-022-01863-7 36210365 PMC9548428

[94] Haque M Ahmed M Nila R Islam S. Classification of human monkeypox disease using deep learning models and attention mechanisms. *Arxiv.* [Preprint]. (2022) 10.48550/arXiv.2211.15459

[95] Pattnaik H Surani S Goyal L Kashyap R. Making sense of monkeypox: a comparison of other poxviruses to the monkeypox. *Cureus.* (2023) 15:e38083. 10.7759/cureus.38083 37252521 PMC10212748

[96] Grant R Nguyen L Breban R. Modelling human-to-human transmission of monkeypox. *Bull World Health Organ.* (2020) 98:638. 10.2471/BLT.19.242347 PMC7463189 33012864

[97] Bunge E Hoet B Chen L Lienert F Weidenthaler H Baer L The changing epidemiology of human monkeypox—a potential threat? a systematic review. *PLoS Neglected Trop Dis.* (2022) 16:e0010141. 10.1371/journal.pntd.0010141 35148313 PMC8870502

[98] Khodakevich L Szczeniowski M Jezek Z Marennikova S Nakano J Messinger D. The role of squirrels in sustaining monkeypox virus transmission. *Trop Geogr Med.* (1987) 39:115–22. 2820094

[99] Berthet N Descorps-Declère S Besombes C Curaudeau M Nkili Meyong A Selekon B Genomic history of human monkey pox infections in the Central African Republic between 2001 and 2018. *Sci Rep.* (2021) 11:13085. 10.1038/s41598-021-92315-8 34158533 PMC8219716

[100] Cho C Wenner H. Monkeypox virus. *Bacteriol Rev.* (1973) 37:1–18. 4349404 10.1128/br.37.1.1-18.1973 PMC413801

[101] Routes C. *Monkeypox Virus.* Boston, MA: Wiley (2009).

[102] Shchelkunov S Totmenin A Babkin I Safronov P Ryazankina O Petrov N Human monkeypox and smallpox viruses: genomic comparison. *FEBS Lett.* (2001) 509:66–70. 11734207 10.1016/S0014-5793(01)03144-1 PMC9533818

[103] Rubins K Hensley L Bell G Wang C Lefkowitz E Brown P Comparative analysis of viral gene expression programs during poxvirus infection: a transcriptional map of the vaccinia and monkeypox genomes. *PLoS One.* (2008) 3:e2628. 10.1371/journal.pone.0002628 18612436 PMC2440811

[104] Kugelman J Johnston S Mulembakani P Kisalu N Lee M Koroleva G Genomic variability of monkeypox virus among humans, Democratic Republic of the Congo. *Emerg Infect Dis.* (2014) 20:232. 10.3201/eid2002.130118 PMC3901482 24457084

[105] Shchelkunov S Totmenin A Safronov P Mikheev M Gutorov V Ryazankina O Analysis of the monkeypox virus genome. *Virology.* (2002) 297:172–94. 12083817 10.1006/viro.2002.1446 PMC9534300

[106] Alkhalil A Hammamieh R Hardick J Ichou M Jett M Ibrahim S. Gene expression profiling of monkeypox virus-infected cells reveals novel interfaces for host-virus interactions. *Virol J.* (2010) 7:1–19. 10.1186/1743-422X-7-173 20667104 PMC2920256

[107] Giorgi F Pozzobon D Di Meglio A Mercatelli D. Genomic characterization of the recent monkeypox outbreak. *Biorxiv.* [Preprint]. (2022) 10.1101/2022.06.01.494368

[108] Hammerschlag Y MacLeod G Papadakis G Sanchez A Druce J Taiaroa G Monkeypox infection presenting as genital rash, Australia, May 2022. *Eurosurveillance.* (2022) 27:2200411. 10.2807/1560-7917.ES.2022.27.22.2200411 PMC9164678 35656835

[109] Cohen-Gihon I Israeli O Shifman O Erez N Melamed S Paran N Identification and whole-genome sequencing of a Monkeypox virus strain isolated in Israel. *Microbiol Resour Announ.* (2020) 9:e1524–1519. 10.1128/MRA.01524-19 32139560 PMC7171222

[110] Otu A Ebenso B Walley J Barceló J Ochu C. Global human monkeypox outbreak: atypical presentation demanding urgent public health action. *Lancet Microbe.* (2022) 3:e554–5. 10.1016/S2666-5247(22)00153-7 35688169 PMC9550615

[111] Abrahim M Guterres A Costa NP Ano B. The emergence of new lineages of the Monkeypox virus could affect the 2022 outbreak. *Biorxiv.* [Preprint]. (2022) 10.1101/2022.07.07.498743

[112] Weaver J Isaacs S. Monkeypox virus and insights into its immunomodulatory proteins. *Immunol Rev.* (2008) 225:96–113. 18837778 10.1111/j.1600-065X.2008.00691.x PMC2567051

[113] Liszewski M Leung M Hauhart R Buller R Bertram P Wang X Structure and regulatory profile of the monkeypox inhibitor of complement: comparison to homologs in vaccinia and variola and evidence for dimer formation. *J Immunol.* (2006) 176:3725–34. 10.4049/jimmunol.176.6.3725 16517741

[114] Lopera J Falendysz E Rocke T Osorio J. Attenuation of monkeypox virus by deletion of genomic regions. *Virology.* (2015) 475:129–38. 25462353 10.1016/j.virol.2014.11.009 PMC4720520

[115] Lansiaux E Jain N Laivacuma S Reinis A. The virology of human monkeypox virus (hMPXV): a brief overview. *Virus Res.* (2022) 32:198932. 10.1016/j.virusres.2022.198932 36165924 PMC9534104

[116] Rambaut A. *Discussion of on-going MPXV genome sequencing.* (2022). Available online at: https://virological.org/t/discussion-of-on-going-MPXV-genomesequencing/802 (accessed on 29 May, 2022).

[117] Perez J. Peculiar evolution of the Monkeypox virus genomes. *Int J Vaccines Vaccin.* (2022) 7:13–6.

[118] Esposito J Knight J. Orthopoxvirus DNA: a comparison of restriction profiles and maps. *Virology.* (1985) 143:230–51. 10.1016/0042-6822(85)90111-4 2998003

[119] Jezek Z Gromyko A Szczeniowski M. Human monkeypox. *J Hyg Epidemiol Microbiol Immunol.* (1983) 27:13–28. 6304185

[120] Bragazzi N Kong J Mahroum N Tsigalou C Khamisy-Farah R Converti M Epidemiological trends and clinical features of the ongoing monkeypox epidemic: a preliminary pooled data analysis and literature review. *J Med Virol.* (2022) 95:e27931. 10.1002/jmv.27931 35692117

[121] Thornhill J Barkati S Walmsley S Rockstroh J Antinori A Harrison L Monkeypox virus infection in humans across 16 Countries — April–June 2022. *N Engl J Med.* (2022) 387:e69. 10.1056/NEJMoa2207323 35866746

[122] Reynolds M McCollum A Nguete B Shongo Lushima R Petersen B. Improving the care and treatment of monkeypox patients in low-resource settings: applying evidence from contemporary biomedical and smallpox biodefense research. *Viruses.* (2017) 9:380. 10.3390/v9120380 29231870 PMC5744154

[123] Nakhaie M Arefinia N Charostad J Bashash D Haji Abdolvahab M Zarei M. Monkeypox virus diagnosis and laboratory testing. *Rev Med Virol.* (2023) 33:e2404. 10.1002/rmv.2404 36331049

[124] Peiró-Mestres A Fuertes I Camprubí-Ferrer D Marcos M Vilella A Navarro M Frequent detection of monkeypox virus DNA in saliva, semen, and other clinical samples from 12 patients, Barcelona, Spain, May to June 2022. *Eurosurveillance.* (2022) 27:2200503. 10.2807/1560-7917.ES.2022.27.28.2200503 35837964 PMC9284919

[125] Girometti N Byrne R Bracchi M Heskin J McOwan A Tittle V Epidemiological characteristics and clinical features of confirmed human monkeypox virus cases in individuals attending a Sexual Health Centre in London, United Kingdom. *SSRN Electr J.* [Preprint]. (2022) 10.2139/ssrn.4125251 PMC9534773 35785793

[126] Martínez J Montalbán E Bueno S Martínez F Juliá A Díaz J Monkeypox outbreak predominantly affecting men who have sex with men, Madrid, Spain, 26 April to 16 June 2022. *Eurosurveillance.* (2022) 27:2200471. 10.2807/1560-7917.ES.2022.27.27.2200471 35801519 PMC9264731

[127] Thornhill J Barkati S Walmsley S Rockstroh J Antinori A Harrison L Monkeypox virus infection in humans across 16 countries—April–June 2022. *N Engl J Med.* (2022) 387:679–91. 35866746 10.1056/NEJMoa2207323

[128] Orviz E Negredo A Ayerdi O Vázquez A Muñoz-Gomez A Monzón S Monkeypox outbreak in Madrid (Spain): clinical and virological aspects. *J Infect.* (2022) 85:412–7. 10.1016/j.jinf.2022.07.005 35830908 PMC9534097

[129] Ježek Z Grab B Szczeniowski M Paluku K Mutombo M. Clinico-epidemiological features of monkeypox patients with an animal or human source of infection. *Bull World Health Organ.* (1988) 66:459. PMC2491168 2844428

[130] Mailhe M Beaumont A Thy M Le Pluart D Perrineau S Houhou-Fidouh N Clinical characteristics of ambulatory and hospitalized patients with monkeypox virus infection: an observational cohort study. *Clin Microbiol Infect.* (2023) 29:233–9. 10.1016/j.cmi.2022.08.012 36028090 PMC9533921

[131] Wang X Lun W. Skin manifestation of human monkeypox. *J Clin Med.* (2023) 12:914. 10.3390/jcm12030914 PMC9918194 36769562

[132] Hughes C McCollum A Pukuta E Karhemere S Nguete B Lushima R Ocular complications associated with acute monkeypox virus infection, DRC. *Int J Infect Dis.* (2014) 21:276–7.

[133] Ogoina D Mohammed A Yinka-Ogunleye A Ihekweazu C. A case of suicide during the 2017 monkeypox outbreak in Nigeria. *IJID Regions.* (2022) 3:226–7. 10.1016/j.ijregi.2022.04.004 35755463 PMC9216383

[134] Badenoch J Conti I Rengasamy E Watson C Butler M Hussain Z Neurological and psychiatric presentations associated with human monkeypox virus infection: a systematic review and meta-analysis. *Eclinicalmedicine.* (2022) 52:101644. 10.1016/j.eclinm.2022.101644 36246957 PMC9533950

[135] Mitjà O Ogoina D Titanji B Galvan C Muyembe J Marks M Monkeypox. *Lancet.* (2023) 401:60–74. 36403582 10.1016/S0140-6736(22)02075-X PMC9671644

[136] Patel A Bilinska J Tam J Fontoura D Mason C Daunt A Clinical features and novel presentations of human monkeypox in a central London centre during the 2022 outbreak: descriptive case series. *BMJ.* (2022) 378:e072410. 10.1136/bmj-2022-072410 35902115 PMC9331915

[137] Català A Clavo-Escribano P Riera-Monroig J Martín-Ezquerra G Fernandez-Gonzalez P Revelles-Peñas L Monkeypox outbreak in Spain: clinical and epidemiological findings in a prospective cross-sectional study of 185 cases. *Br J Dermatol.* (2022) 187:765–72. 10.1111/bjd.21790 35917191

[138] Tarín-Vicente E Alemany A Agud-Dios M Ubals M Suñer C Antón A Clinical presentation and virological assessment of confirmed human monkeypox virus cases in Spain: a prospective observational cohort study. *Lancet.* (2022) 400:661–9. 10.1016/S0140-6736(22)01436-2 35952705 PMC9533900

[139] Huhn G Bauer A Yorita K Graham M Sejvar J Likos A Clinical characteristics of human monkeypox, and risk factors for severe disease. *Clin Infect Dis.* (2005) 41:1742–51. 16288398 10.1086/498115

[140] Ježek Z Szczeniowski M Paluku K Mutombo M. Human monkeypox: clinical features of 282 patients. *J Infect Dis.* (1987) 156:293–8. 10.1093/infdis/156.2.293 3036967

[141] Anderson M Frenkel L Homann S Guffey J. A case of severe monkeypox virus disease in an American child: emerging infections and changing professional values. *Pediatr Infect Dis J.* (2003) 22:1093–6. 10.1097/01.inf.0000101821.61387.a5 14688573

[142] Sale T Melski J Stratman E. Monkeypox: an epidemiologic and clinical comparison of African and US disease. *J Am Acad Dermatol.* (2006) 55:478–81. 10.1016/j.jaad.2006.05.061 16908354 PMC9629018

[143] Moore M Zahra F. *Monkeypox.* Tampa, FL: StatPearls Publishing (2021). 34662033

[144] Doshi R Guagliardo S Doty J Babeaux A Matheny A Burgado J Epidemiologic and ecologic investigations of monkeypox, Likouala Department, Republic of the Congo, 2017. *Emerg Infect Dis.* (2019) 25:273. 10.3201/eid2502.181222 30666937 PMC6346463

[145] Ogoina D Izibewule J Ogunleye A Ederiane E Anebonam U Neni A The 2017 human monkeypox outbreak in Nigeria—report of outbreak experience and response in the Niger Delta University Teaching Hospital, Bayelsa State, Nigeria. *PLoS One.* (2019) 14:e0214229. 10.1371/journal.pone.0214229 30995249 PMC6469755

[146] Jezek Z Grab B Szczeniowski M Paluku K Mutombo M. Human monkeypox: secondary attack rates. *Bull World Health Organ.* (1988) 66:465–70. 2844429 PMC2491159

[147] Stagles M Watson A Boyd J More I McSeveney D. The histopathology and electron microscopy of a human monkeypox lesion. *Trans R Soc Trop Med Hyg.* (1985) 79:192–202. 2988159 10.1016/0035-9203(85)90333-5

[148] Fenner F Henderson D Arita I Jezek Z Ladnyi I. Human monkeypox and other poxvirus infections of man. In: Fenner F Henderson D Arita I Jezek Z Ladnyi I editors. *Smallpox and its Eradication.* Geneva: World Health Organization (1988). p. 1287–320.

[149] Altindis M Puca E Shapo L. Diagnosis of monkeypox virus – An overview. *Travel Med Infect Dis.* (2022) 50:102459. 10.1016/j.tmaid.2022.102459 PMC9534096 36109000

[150] Bayer-Garner I. Monkeypox virus: histologic, immunohistochemical and electron-microscopic findings. *J Cutaneous Pathol.* (2005) 32:28–34. 10.1111/j.0303-6987.2005.00254.x 15660652

[151] Marennikova S Nagieva F Matsevich G Shelukhina E Khabakhpasheva A Platonova G. Monoclonal antibodies to monkey pox virus: preparation and application. *Acta Virol.* (1988) 32:19–26. 2897768

[152] Singhal T Kabra S Lodha R. Monkeypox: a review. *Indian J Pediatr.* (2022) 89:955–60. 10.3390/v14102155 35947269 PMC9363855

[153] Kulesh D Loveless B Norwood D Garrison J Whitehouse C Hartmann C Monkepox virus detection in rodents using real-time 3′-minor groove binder TaqMan^®^ assays on the Roche LightCycler. *Lab Invest.* (2004) 84:1200–8. 10.1038/labinvest.3700143 15208646 PMC9827366

[154] Li Y Olson V Laue T Laker M Damon I. Detection of monkeypox virus with real-time PCR assays. *J Clin Virol.* (2006) 36:194–203. 10.1016/j.jcv.2006.03.012 16731033 PMC9628957

[155] Olson V Laue T Laker M Babkin I Drosten C Shchelkunov S Real-time PCR system for detection of orthopoxviruses and simultaneous identification of smallpox virus. *J Clin Microbiol.* (2004) 42:1940–6. 10.1128/JCM.42.5.1940-1946.2004 15131152 PMC404623

[156] Shchelkunov S Shcherbakov D Maksyutov R Gavrilova E. Species-specific identification of variola, monkeypox, cowpox, and vaccinia viruses by multiplex real-time PCR assay. *J Virol Methods.* (2011) 175:163–9. 10.1016/j.jviromet.2011.05.002 21635922 PMC9628778

[157] Fleischauer A Kile J Davidson M Fischer M Karem K Teclaw R Evaluation of human-to-human transmission of monkeypox from infected patients to health care workers. *Clin Infect Dis.* (2005) 40:689–94. 10.1086/427805 15714414

[158] Petersen E Kantele A Koopmans M Asogun D Yinka-Ogunleye A Ihekweazu C Human monkeypox: epidemiologic and clinical characteristics, diagnosis, and prevention. *Infect Dis Clin.* (2019) 33:1027–43. 10.1016/j.idc.2019.03.001 30981594 PMC9533922

[159] Roess A Monroe B Kinzoni E Gallagher S Ibata S Badinga N Assessing the effectiveness of a community intervention for monkeypox prevention in the Congo basin. *PLoS Neglected Trop Dis.* (2011) 5:e1356. 10.1371/journal.pntd.0001356 22028942 PMC3196471

[160] Adalja A Inglesby T. *A novel international monkeypox outbreak.* Philadelphia: American College of Physicians (2022). 10.7326/M22-1581 35605243

[161] Meyer H. *Summary report on first, second and third generation smallpox vaccines.* Geneva: World Health Organization (2013).

[162] Jordan R Leeds J Tyavanagimatt S Hruby D. Development of ST-246^®^ for treatment of poxvirus infections. *Viruses.* (2010) 2:2409–35. 10.3390/v2112409 21994624 PMC3185582

[163] Baker R Bray M Huggins J. Potential antiviral therapeutics for smallpox, monkeypox and other orthopoxvirus infections. *Antiviral Res.* (2003) 57:13–23. 10.1016/s0166-3542(02)00196-1 12615299 PMC9533837

[164] Chavda V Vora L Apostolopoulos V. Monkeypox: a new face of outbreak. *Expert Rev Vaccin.* (2022) 21:1537–40. 10.1080/14760584.2022.2113515 35968670 PMC9491104

[165] Grabenstein J Winkenwerder W. US military smallpox vaccination program experience. *JAMA.* (2003) 289:3278–82. 10.1001/jama.289.24.3278 12824209

[166] Okyay R Bayrak E Kaya E Şahin A Koçyiğit B Taşdoğan A Another Epidemic in the Shadow of Covid 19 Pandemic: a Review of Monkeypox. *Proteins.* (2022) 7:10. 10.1016/j.jiph.2023.05.013 37269693 PMC10182868

[167] Jabeen C Umbreen G. Monkeypox transmission, need and treatment of humans with an antiviral drug. *Int J Soc Sci Manag.* (2017) 4:77–9. 10.3390/ijms232415941 36555584 PMC9784635

[168] Berhanu A Prigge J Silvera P Honeychurch K Hruby D Grosenbach D. Treatment with the smallpox antiviral tecovirimat (ST-246) alone or in combination with ACAM2000 vaccination is effective as a postsymptomatic therapy for monkeypox virus infection. *Antimicrob Agents Chemother.* (2015) 59:4296–300. 10.1128/AAC.00208-15 25896687 PMC4468657

[169] Mucker E Goff A Shamblin J Grosenbach D Damon I Mehal J Efficacy of tecovirimat (ST-246) in nonhuman primates infected with variola virus (Smallpox). *Antimicrob Agents Chemother.* (2013) 57:6246–53. 10.1128/AAC.00977-13 24100494 PMC3837858

[170] Isaacs S. Literature review current through: May 2022. Alphen aan den Rijn: Wolters Kluwer (2022).

